# Marginal superiority of maize: an indicator for density tolerance under high plant density

**DOI:** 10.1038/s41598-020-72435-3

**Published:** 2020-09-21

**Authors:** Guangzhou Liu, Wanmao Liu, Yunshan Yang, Xiaoxia Guo, Guoqiang Zhang, Jian Li, Ruizhi Xie, Bo Ming, Keru Wang, Peng Hou, Shaokun Li

**Affiliations:** 1 Key Laboratory of Crop Physiology and Ecology, Institute of Crop Sciences, Chinese Academy of Agricultural Sciences, Ministry of Agriculture and Rural Affairs, Beijing, 100081 China; 2 The Key Laboratory of Oasis Eco-Agriculture, Xinjiang Production and Construction Corps, College of Agronomy, Shihezi Univerisy, Shihezi, 832000 China; 3 State Key Laboratory of Cotton Biology/Institute of Cotton Research, Chinese Academy of Agricultural Sciences, Anyang, 455000 China

**Keywords:** Plant development, Plant stress responses

## Abstract

Marginal superiority is a common phenomenon in crops, and is caused by the competitiveness of individual plant for resources and crop adaptability to crowded growth conditions. In this study, in order to clarify the response of marginal superiority to maize morphology and plant-density tolerance, field experiments without water and nutrition stress were conducted at Qitai Farm in Xinjiang, China, in 2013–2014 and 2016–2019. The results showed that no more than three border rows of all the cultivars had marginal superiority under high density, about 90% of all the cultivars had no more than two border row that had marginal superiority and a significant negative correlation was observed between marginal superiority and population grain yield (first border row: y = − 2.193x + 213.9, p < 0.05; second border row: y = − 2.076x + 159.2, p < 0.01). Additionally, marginal superiority was found to have a significant positive relationship with plant density (first border row: y = 6.049x + 73.76, p < 0.01; second border row: y = 1.88x + 95.41, p < 0.05) and the average leaf angle above the ear (first border row: y = 2.306x + 103.1, p < 0.01). These results indicated that the smaller the leaf angle above the ear, the weaker the marginal superiority and the higher the grain yield. It suggests that the magnitude of marginal superiority in the border rows can be an indicator for plant-density tolerance under high density. What’s more, cultivars with small leaf angle above the ear can be selected to weaken the marginal superiority and improve grain yield under high plant density. Conversely, cultivars with a large leaf angle above the ear can be selected to achieve higher individual yield in intercropping systems with no more than four rows alternated with other crops.

## Introduction

Selecting the optimum plant population allows maize to intercept and use solar radiation more efficiently, which contributes to a remarkable increase in grain yield potential^1–3^. However, excessively high plant density may cause crowding stress and poor light conditions in the crop population, which can reduce the grain yield^4–7^, possibly due to the increased competitiveness of individual plants for resources^8,9^. Previous studies have shown that the competitiveness is weak among individuals in high-yield crop populations^10–12^. However, it has also been shown that individuals enjoy more resources in the border rows, which results in higher individual yield^13–15^; this phenomenon is referred to as marginal superiority^16,17^.

Marginal superiority is always considered in cultivar test experiments to precisely assess the grain yield^18,19^, and this phenomenon is also used in intercropping systems to improve crop yield^14,20–23^. The magnitude of marginal superiority reflects the plant-density tolerance of maize cultivars^24,25^, that is, the adaptability and competitiveness of crops under high plant density. The genetic improvement of maize grain yield was associated with the increased tolerant to stress^26^. The plant-density tolerance may be related to plant height, ear height, or leaf angle^11,12^. Previous studies have shown that these morphological traits can contribute to high grain yield^6,27^, such as lower plant height and ear height, and small leaf angle, which can weaken the competitiveness of individual plants and allow each individual plant to use resources more effectively^12,28^. Therefore, both the plant density and plant type may affect the marginal superiority. However, most previous studies were conducted under traditional low plant density and low yield, and the interaction between marginal superiority and plant-density tolerance, plant type, and grain yield have rarely been studied under higher plant density.

In previous studies, through more than 10 years of field investigation at Qitai Farm in Xinjiang, China, we observed significant differences in grain yields^6,7,29^ and different individual yield by visual observation in the border rows between different high-yield maize cultivars under high plant density. Based on the results of these studies, we hypothesized that the marginal superiority in the border rows could be used as an indicator of plant-density tolerance under high plant density. To verify this hypothesis, in the present study, 6 years of experiments were conducted under high plant density. The main objectives were to clarify: (1) how many rows have marginal superiority under high plant density and high grain yield; (2) the relationship between marginal superiority and population density and grain yield; (3) the relationship between marginal superiority and plant type and grain yield.

## Results

### The population grain yield and individual yield in different border rows of different cultivars

During all the experimental years, the final grain yields of the selected cultivars were all more than 11.0 Mg ha^−1^ (Table S1). The results of the ANOVA between individual yields in different border rows showed that there was no significant difference after the third border row for any cultivars in any experimental year, and about 90% of all the cultivars that there was no significant difference after the second border row. Therefore, it was considered that no more than the outermost three border rows had marginal superiority, and based on this a further analysis was made, which was described as follows. The average individual yield from the fourth to the sixth border row was regarded as the individual yield of the inner row, and then the individual yield of the first border row, second border row, and third border row were divided by this mean value, respectively, to calculate the marginal superiority (Eq. 1) in each of these rows. The marginal superiorities of the first, second, and third border rows are denoted as Ratio-1, Ratio-2, and Ratio-3, respectively.

### Relationship between marginal superiority, plant density, leaf angle above and grain yield

Population grain yield was significantly negatively correlated with the superiority of the first border row and second border row, that is, the superiority of the first and second border rows decreased with increasing yield (Table 1). Furthermore, the plant density was significantly positively correlated with the superiority of the first border row and second border row, that is, the superiority of the first border row and second border row increased with increasing plant density. Moreover, the leaf angle above the ear was significantly positively correlated with the superiority of the first border row, meaning that cultivars with an erect plant type had weak superiority in the first border row. However, there was no significant correlation between the leaf angle above the ear and the superiority of the second border row. Additionally, there was no significant correlation between the superiority of the third border row and population grain yield, plant density, or the leaf angle above the ear.

**Table 1:** Results of the linear regression analysis between the marginal superiority and population grain yield, plant density, and leaf angle.

Superiority	Grain yield			Plant density			Leaf angle above ear		
	Equation	R^2^	p value	Equation	R^2^	p value	Equation	R^2^	p value
Ratio-1	Y = − 2.193x + 213.9	0.0576*	0.0205	Y = 6.049x + 73.76	0.1333**	0.0000	Y = 2.306x + 103.1	0.1846**	0.0080
Ratio-2	Y = − 2.076x + 159.2	0.0800**	0.0060	Y = 1.88x + 95.41	0.0445*	0.0224	–	ns	0.1370
Ratio-3	–	ns	0.0920	–	ns	0.2368	–	ns	0.0633

Ratio-1, Ratio-2, and Ratio-3 denote the marginal superiorities of the first, second, and third border rows, respectively.

*Significance at the p < 0.05 level.

**Significance at the p < 0.01 level; ns denotes no significant difference.

**Figure 1:**
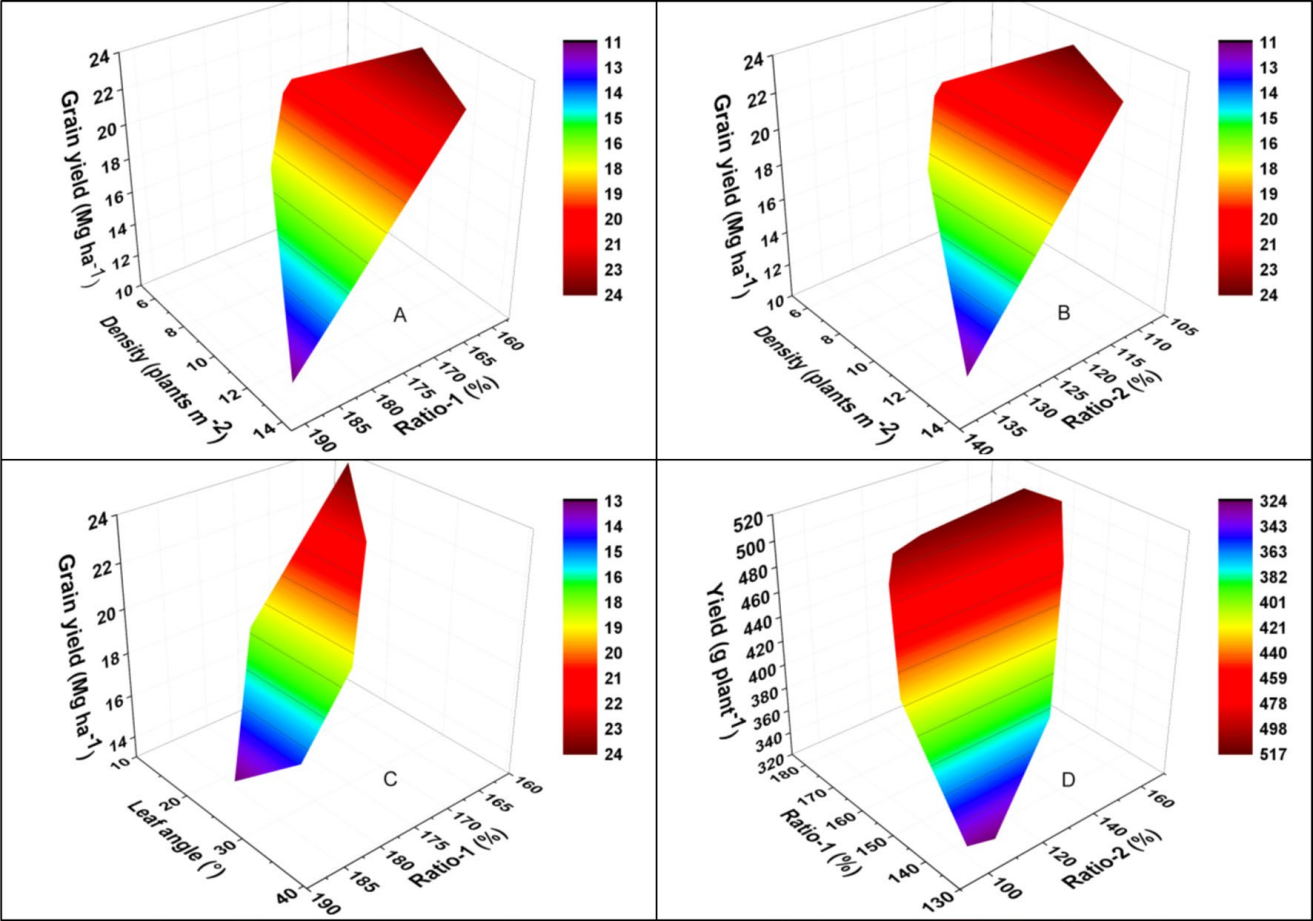
The interactive effects of marginal superiority, plant density (**A**, **B**), leaf angle above the ear (**C**) on the grain yield, and the effects of the marginal superiority on the total individual yield per plant in the first and second border row (**D**). Ratio-1 is the marginal superiority of the first border row and Ratio-2 is the marginal superiority of the second border row.

With increasing plant density, the population grain yield first increased and then decreased (Fig. 1A,B), with the highest grain yield being obtained for a density of 10.5 plants m^−2^ (Experiment II, data not shown). Moreover, the marginal superiority of the first border row (Fig. 1A) and second border row (Fig. 1B) both increased with increasing plant density, however, they both decreased with increasing population grain yield. Therefore, when a high yield is obtained under a high plant density, maize cultivars should have weak marginal superiority to improve their plant-density tolerance.

The population grain yield increased with decreasing leaf angle above the ear, however the marginal superiority of the first border row decreased with decreasing leaf angle above the ear (Fig. 1C). This can be attributed to the fact that, with decreasing leaf angle above the ear, the plant type is more erect, which may weaken the competitiveness between individuals in the population and therefore also weaken marginal superiority, resulting in the increase of grain yield. On the contrary, with increasing leaf angle above the ear, the competition for resources between individuals in the population may increase, which may consequently increase the marginal superiority and decrease the grain yield. In addition, when the leaf angle above ear was between 13.9° and 20.1°, grain yield can be obtained more than 19.0 Mg ha^−1^ steadily under high density.

The total individual grain yields in the border rows increased with increasing marginal superiority of the first border row and second border row, respectively (Fig. 1D). In other words, strong marginal superiorities of the first border row and second border row were associated with a higher total individual yield in the border rows.

## Discussion

Increasing plant density is an important way to improve maize grain yield, as was verified in our previous study^7,29,30^. In the present study, the grain yields were universally more than 11.0 Mg ha^−1^ (Table S1), and the highest obtained yield was 23.9 Mg ha^−1^, which was obtained at a density of 10.5 plants m^−2^ (data not shown, Experiment II). Grain yield varied among different years mainly due to different weather conditions (Table 2). However, previous studies have shown that high plant density may cause poor growth conditions in the population^6,7^ and enhance intraspecific competition^9^; this can increase the individual yield in the border rows relative to the individual yield in the inner rows^14,15^, a phenomenon referred to as marginal superiority. Therefore, such marginal superiority can be expected to increase with increasing plant density, as was observed in the present study (Table 1, Fig. 1A,B).

**Table 2:** Mean daily maximum temperature (T_max_), mean daily minimum temperature (T_min_), mean daily solar radiation and total precipitation (Pre) during the maize growing season at Qitai Farm in 2013–2014, 2016–2019.

Year	T_max_ (°C)	T_min_ (°C)	Solar radiation (MJ m^−2^ day^−1^)	Pre (mm)
2013	25.2	9.6	19.6	160.6
2014	25.0	9.7	18.0	138.7
2016	27.9	13.3	19.0	176.3
2017	26.0	13.2	19.2	172.4
2018	24.4	10.1	19.4	221.0
2019	25.8	11.4	19.6	138.5

Marginal superiority in the border rows reflects plant-density tolerance of maize cultivars^25^. Cultivars with higher grain yield generally have weak competitiveness^28^ and weak marginal superiority^24^; accordingly, cultivars with a higher grain yield can be expected to have weak marginal superiority, as was found in the present study (Table 1, Fig. 1A–C). The observed relationships between marginal superiority, plant density, and grain yield (Fig. 1A,B) indicated that excessively high plant density showed high marginal superiority but it was adverse to grain yield. Therefore, the present results suggested that the magnitude of marginal superiority could be used as an indicator for evaluating the plant-density tolerance, which was consistent with the findings of Jiao et al.^25^. Previous studies showed that cultivars with weak competitiveness might have shorter plant height, shorter ear height, and a more compact plant type^11,12^. In the present study, it was found that the leaf angle above the ear was positively correlated with marginal superiority (Table 1, Fig. 1C), which indicated that compact cultivars had more reasonable light distribution and thus weak competitiveness and weak marginal superiority^12,23,28^, which might have contributed to their high grain yields^30^.

In this study, it was found that the superiorities of the first and second border rows were significantly correlated with the population grain yield and plant density, especially the superiority of the first border row. This indicated that, in the studied modern maize cultivars, marginal superiority existed in no more than the outermost three border rows under high plant density and yield level, which was consistent with previous finding^31^. Moreover, the magnitude of marginal superiority was found to be different between different cultivars which was also consistent with previous studies^32,33^. However, a previous study on rice showed that only one border row had marginal superiority^33^; this difference in number of rows with marginal superiority might be due to that different species may have different magnitude of marginal superiority and may be attributed to the difference in stem height.

Intercropping systems are widely used in many countries, especially in China^14,34^. Such systems can improve grain yield by increasing marginal effects^20–22^. In this study, a significant positive relationship (data was not shown) was found between total individual yield and marginal superiority in the first border row and second border row (Fig. 1D). This suggested that maize cultivars with strong marginal superiority would produce higher individual yields and could be used in intercropping systems with no more than four rows alternated with other crops, which was consistent with the findings of previous studies^8,14,21^.

## Conclusion

With the analysis of the relationship between marginal superiority and grain yield, plant density, and plant type in modern maize hybrids, it was found that the marginal superiority was significantly affected by the population density and leaf angle above the ear, and was closely related to population grain yield. Specifically, the higher the population density, the stronger the marginal superiority, however the weaker the marginal superiority, the higher the grain yield. Additionally, it was found that the smaller the leaf angle above the ear, the weaker the marginal superiority and the higher the grain yield. Only one or two border rows had marginal superiority for about 90% of the tested modern maize hybrids under high density. Furthermore, it was also observed that stronger marginal superiority was associated with higher total individual yield in the first border row and the second border row. Therefore, the magnitude of marginal superiority in the border rows can be an indicator for plant-density tolerance under high plant density.

## Materials and methods

### Site and experiment design

Field experiments were conducted in 2013–2014 and 2016–2019 at the Qitai Farm (43° 49′ 27″ N, 89° 48′ 22″ E) in Xinjiang, China, using a traditional alternating narrow–wide row planting pattern, where the width of the narrow row was 0.4 m and the width of the wide row was 0.7 m, with an average row spacing of 0.55 m. The mean daily maximum temperature (T_max_), mean daily minimum temperature (T_min_), mean daily solar radiation, total precipitation (Pre) during the maize growing season in the 6 experimental years were shown in the Table 2. As shown in Table 2, T_max_ and T_min_ in 2016 and 2017 were higher than other years. Daily solar radiation in 2014 was the lowest during the experimental years. Pre in 2014 and 2019 were lower than other years.

Experiment I: In order to study the marginal superiority of different maize hybrids under high density, field experiments were conducted in 2013–2014, 2016–2019. In 2013, a total of 22 maize cultivars were planted at a density of 13.5 plants m^−2^. In 2014, a total of eight maize cultivars were planted, with a density of 13.5 plants m^−2^ used for six of these cultivars and a density of 12.0 plants m^−2^ used for the remaining two cultivars. In 2016, a total of seven cultivars were planted at a density of 13.5 plants m^−2^. In 2017, a total of 14 cultivars were planted at a density of 13.5 plants m^−2^. In 2018, a total of 11 cultivars were planted at a density of 13.5 plants m^−2^. In 2019, a total of 23 cultivars were planted, with a density of 13.5 plants m^−2^ used for 17 of these cultivars and a density of 10.5 plants m^−2^ used for the remaining six cultivars. The experimental design was a completely randomized design with three replications. Each plot was 19.8 m in width and 21 m in length, and there were 36 rows in each plot.

Experiment II: In order to study the relationship between plant density and marginal superiority, field experiments were conducted in 2017 and 2019. In 2017, maize cultivars liangyu66 (LY66) and denghai 618 (DH618) were planted at densities of 7.5, 10.5, and 13.5 plants m^−2^, and cultivars xianyu 335 (XY335) and zhengdan (ZD958) were planted at densities of 6.0, 9.0, and 12.0 plants m^−2^. In 2019, maize cultivars DH618, SC704, and MC670 were planted at densities of 7.5, 10.5, 12.0, and 13.5 plants m^−2^. The experimental design was a completely randomized design with three replications. Each plot was 11 m in width and 10 m in length, and there were 22 rows in each plot.

In both of the two experiments, seeds were planted in mid-April and plants were harvested in mid-October. Sufficient fertilizer and water were applied to prevent nutrient and water stress, and the field management was the same as that in our previous study^7^.

### Sampling and measurement

In Experiment I, the leaf angle (i.e., the angle between the stem and the line from the leaf base point to the zenith of the leaf) at the silking stage was measured for 37 cultivars for the periods 2013–2014 and 2016–2019. Three successive plants in each center plot were selected to measure the leaf angle, and the angle was measured for all the leaves above the ear (there was only one ear beared per plant under plant density of more than 10.5 plants m^−2^).

**Figure 2:**
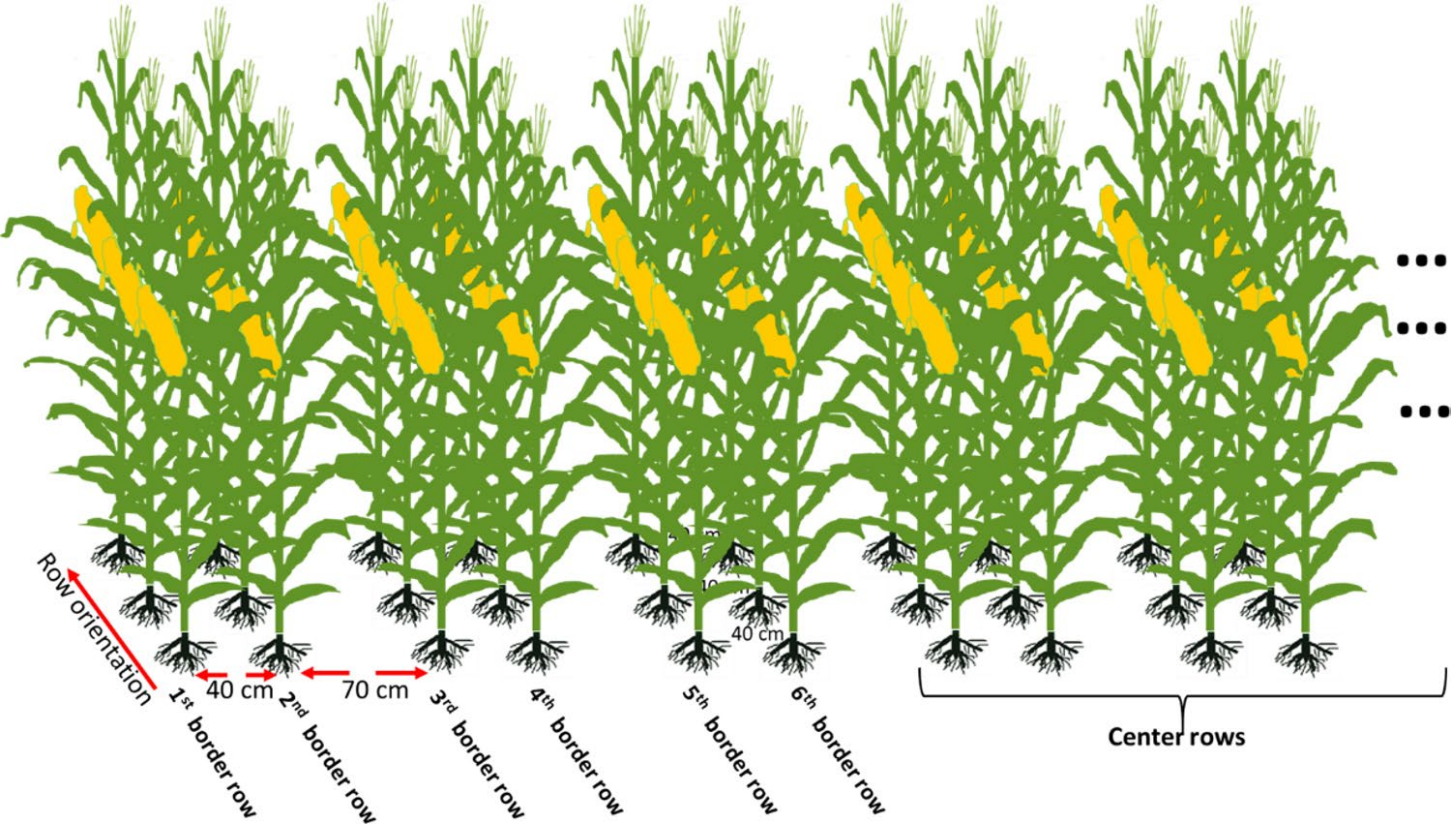
Schematic diagram of the border rows in a plot.

At physiological maturity, the ears of 5–10 successive plants were harvested in each border row which contained sufficient plants (from the first border row to the sixth border row) in the same side of each plot in Experiment I and Experiment II (Fig. 2). In 2016, a total of four border rows (from the first border row to the fourth border row) were sampled in Experiment I. All the ears were artificially threshed in order to calculate the individual yield per plant. Additionally, grains were also harvested from a length of 10 m in 12 successive rows in the center of each plot in Experiment I and from a length of 5 m in six successive rows in the center of each plot in Experiment II. The individual and population grain yield were calculated at 14.0% grain moisture content, which was measured with a portable moisture meter (PM8188, Kett Electric Laboratory, Tokyo, Japan).

The marginal superiority in the border row was calculated as follows:

1 $${\text{Marginal superiority}} = {\text{individual yield of border row/individual yield of the inner row}} \times 100\%$$

### Statistical analysis

Linear regression analysis was performed to examine the relationship between marginal superiority and grain yield, plant density, and average leaf angle above the ear, and analysis of variance (ANOVA) was used to test the significance of differences in individual yield per plant in different border rows. Both linear regression and ANOVA were performed using the SPSS v. 21.0 software (IBM Inc., Armonk, NY, USA).

## Supplementary information

Supplementary Table S1.
