# Supplementary material for: Marginal superiority of maize: an indicator for density tolerance under high plant density
Source: Sci Rep. 2020 Sep 21;10:15378. doi: 10.1038/s41598-020-72435-3 (PMC7505976; doi:10.1038/s41598-020-72435-3)
Supplement: Supplementary file 1 — Supplementary Table S1. [file 41598_2020_72435_MOESM1_ESM.docx]

**Marginal superiority of maize-an indicator for density tolerance under high plant density**

Guangzhou Liu ^1^, Wanmao Liu ^2^, Yunshan Yang ^2^, Xiaoxia Guo ^2^, Guoqiang Zhang ^1^, Jian Li ^3^, Ruizhi Xie ^1^, Bo Ming ^1^, Keru Wang ^1^, Peng Hou ^1^*, Shaokun Li ^1^*

1 Key Laboratory of Crop Physiology and Ecology, Institute of Crop Sciences, Chinese Academy of Agricultural Sciences, Ministry of Agriculture and Rural Affairs, Beijing 100081, China

2 The Key Laboratory of Oasis Eco-agriculture, Xinjiang Production and Construction Corps, College of Agronomy, Shihezi Univerisy, Shihezi 832000, China

3 State Key Laboratory of Cotton Biology/ Institute of Cotton Research, Chinese Academy of Agricultural Sciences, Anyang 455000, China

*Corresponding authors: Shaokun Li, Professor, Tel: 0086-10-82108891, Fax: 0086-10-82108891, E-mail: lishaokun@caas.cn; Peng Hou, Professor, Tel: 0086-10-82108595, Fax: 0086-10-82108595, E-mail: houpeng@caas.cn

**Table S1.** Population grain yield and mean yield per plant in different border rows (B1-B6) in the six experimental years for different maize cultivars and plant densities. Means in the same row not sharing any letters are significantly different at the 5% significance level.

| **Year** | **Cultivar** | **Density**  **(plants m^-2^)** | **Yield**  **(Mg ha^-1^)** | **Individual yield (g plant^-1^)** | | | | | |
| --- | --- | --- | --- | --- | --- | --- | --- | --- | --- |
|  |  |  |  | **B1** | **B2** | **B3** | **B4** | **B5** | **B6** |
| 2013 | SD609 | 13.5 | 17.0 | 323.8a | 193.7b | 194.5b | 189.1b | 193.2b | 171.5b |
|  | XY335 | 13.5 | 20.8 | 323.7a | 186.7b | 177.5b | 158.8b | 161.7b | 152.7b |
|  | JD73 | 13.5 | 21.3 | 320.1a | 215.0b | 197.9bc | 189.4bc | 165.9bc | 158.5c |
|  | DH605 | 13.5 | 20.2 | 314.1a | 175.3b | 175.9b | 173.5b | 179.9b | 155.2b |
|  | ZD958 | 13.5 | 19.8 | 311.5a | 202.4b | 170.7bc | 135.2c | 138.7c | 138.3c |
|  | ZD909 | 13.5 | 19.6 | 298.2a | 218.5b | 178.8c | 142.1c | 143.3c | 130.7c |
|  | JH3055 | 13.5 | 19.5 | 286.7a | 182.5b | 155.4b | 154.7b | 152.4b | 147.3b |
|  | M751 | 13.5 | 15.1 | 275.7a | 217.5b | 179.3bc | 154.2c | 147.9c | 144.1c |
|  | LY66 | 13.5 | 20.8 | 270.0a | 231.1b | 144.1c | 132.1c | 138.1c | 138.1c |
|  | JH1029 | 13.5 | 17.3 | 269.7a | 193.3b | 155.9c | 126.0d | 117.6d | 112.2d |
|  | SD606 | 13.5 | 14.2 | 266.4a | 195.2b | 180.1b | 123.9c | 120.4c | 124.7c |
|  | NH101 | 13.5 | 20.0 | 261.0a | 159.6b | 141.6b | 134.8b | 139.8b | 144.7b |
|  | KWS3564 | 13.5 | 21.5 | 254.7a | 194.4b | 153.0bc | 143.6c | 146.0c | 143.5c |
|  | PA169 | 13.5 | 14.6 | 251.5a | 199.8b | 184.7bc | 159.4bc | 153.2c | 151.0c |
|  | YD606 | 13.5 | 16.6 | 241.8a | 224.6ab | 204.7ab | 196.1ab | 184.2b | 183.2b |
|  | M753 | 13.5 | 13.9 | 241.1a | 193.9b | 155.9bc | 150.8bc | 124.2c | 125.5c |
|  | SD3622 | 13.5 | 11.3 | 232.0a | 185.5b | 164.6bc | 141.7c | 138.1c | 133.0c |
|  | LY99 | 13.5 | 18.5 | 228.7a | 212.5a | 166.1b | 164.5b | 168.0b | 164.3b |
|  | J9913 | 13.5 | 19.3 | 221.1a | 181.5b | 141.6c | 137.1c | 129.2c | 131.9c |
|  | SD3621 | 13.5 | 19.7 | 221.1a | 187.7a | 184.2a | 112.4b | 107.8b | 108.4b |
|  | LM33 | 13.5 | 18.6 | 222.7a | 162.6b | 119.2bc | 115.6c | 113.9c | 113.2c |
|  | LD565 | 13.5 | 18.0 | 193.1a | 147.5b | 128.1bc | 139.5bc | 121.1c | 126.2bc |
| 2014 | LM33 | 12.0 | 13.2 | 219.1a | 146.8b | 132.5bc | 137.6bc | 115.3c | 129.1bc |
|  | DK517 | 13.5 | 14.9 | 227.7a | 149.1b | 130.6b | 127.6b | 95.4c | 95.3c |
|  | LY66 | 12.0 | 15.0 | 245.3a | 215.6b | 129.5c | 124.0c | 126.9c | 115.1c |
|  | M753 | 13.5 | 15.7 | 260.1a | 155.0b | 153.4b | 146.7bc | 141.5bc | 119.8c |
|  | DL1101 | 13.5 | 16.1 | 183.5a | 146.1b | 141.9b | 140.4b | 137.3b | 138.7b |
|  | M753 | 12.0 | 16.5 | 270.3a | 176.2b | 155.2b | 165.8b | 161.5b | 171.4b |
|  | XY19 | 12.0 | 17.7 | 210.3a | 173.3b | 171.0b | 159.9b | 159.0b | 154.6b |
|  | DK517 | 12.0 | 18.2 | 264.9a | 204.1b | 189.1bc | 175.9bc | 153.7c | 157.6c |
|  | DH618 | 12.0 | 19.3 | 270.2a | 205.4b | 177.2bc | 164.2c | 162.5c | 168.5bc |
|  | DH618 | 13.5 | 21.1 | 286.2a | 222.2b | 196.5c | 183.4cd | 183.6cd | 168.9d |
| 2016 | M753 | 13.5 | 18.9 | 287.5a | 206.88b | 163.67bc | 143.92c | - | - |
|  | M751 | 13.5 | 20.7 | 259.94a | 194.13b | 172.98b | 170.56b | - | - |
|  | DH618 | 13.5 | 18.3 | 252.78a | 159.85b | 149.46c | 149.38c | - | - |
|  | DH1119 | 13.5 | 20.6 | 265.2a | 180.14b | 178.29b | 165.74b | - | - |
|  | XY335 | 13.5 | 17.1 | 300.87a | 230.35b | 174.69c | 167.85c | - | - |
|  | KWS3564 | 13.5 | 19.8 | 264.88a | 177.22b | 182.18b | 148.42c | - | - |
|  | YY274 | 13.5 | 15.5 | 262.80a | 172.69b | 136.92c | 125.31c | - | - |
| 2017 | LC825 | 13.5 | 16.8 | 183.2a | 150.6b | 149.1b | 144.0bc | 135.8c | 131.3c |
|  | JYY02 | 13.5 | 17.7 | 257.0a | 236.9a | 229.6a | 244.8a | 222.8a | 187.2b |
|  | JZ58 | 13.5 | 17.7 | 206.3a | 159.9b | 144.4b | 152.5b | 153.9b | 149.0b |
|  | ZY501 | 13.5 | 18.6 | 235.2a | 209.7b | 198.5b | 192.5bc | 173.0c | 172.0c |
|  | DH1786 | 13.5 | 19.0 | 175.3a | 169.6ab | 162.7bc | 159.3bc | 152.9bc | 144.9c |
|  | SD620 | 13.5 | 19.2 | 186.1a | 169.2ab | 169.8ab | 171.4ab | 158.8b | 160.6b |
|  | HY187 | 13.5 | 19.9 | 230.0a | 195.8b | 175.7bc | 177.7bc | 172.7c | 184.5bc |
|  | XY47 | 13.5 | 20.3 | 263.1a | 210.3b | 184.7c | 179.5c | 207.4bc | 191.5bc |
|  | M751 | 13.5 | 20.7 | 251.7a | 227.5b | 213.6bc | 222.2bc | 206.4bc | 203.4c |
|  | LC808 | 13.5 | 21.1 | 268.5a | 221.0b | 220.1b | 200.7c | 169.1d | 196.9c |
|  | YQ909 | 13.5 | 21.1 | 209.0a | 177.1b | 162.6b | 175.6b | 160.6b | 161.7b |
|  | ZY1317 | 13.5 | 22.2 | 191.3a | 194.3a | 193.0a | 174.6a | 180.2a | 147.7b |
|  | DH1796 | 13.5 | 22.4 | 192.1a | 155.4b | 155.0b | 161.6b | 146.6b | 152.2b |
|  | MC670 | 13.5 | 22.8 | 276.4a | 188.6b | 162.2c | 179.1bc | 177.8bc | 167.9bc |
| 2018 | LD575 | 13.5 | 22.4 | 324.2a | 252.1ab | 253.2ab | 220.0b | 221.8b | 219.9b |
|  | DH551 | 13.5 | 21.7 | 254.7a | 250.7a | 244.6a | 257.5a | 238.5a | 202.3b |
|  | NH106 | 13.5 | 21.0 | 282.4a | 270.1a | 267.9a | 261.6ab | 252.1ab | 223.0b |
|  | DH177 | 13.5 | 21.0 | 268.5a | 169.7b | 166.8b | 163.7b | 161.1b | 149.2b |
|  | KWS9384 | 13.5 | 15.4 | 266.0a | 216.4b | 189.5bc | 191.3bc | 166.7c | 197.9bc |
|  | DH1786 | 13.5 | 15.6 | 182.6a | 146.1b | 147.2b | 146.7b | 152.5b | 139.1b |
|  | NH178 | 13.5 | 18.1 | 262.9a | 259.8a | 209.4ab | 217.7b | 191.9b | 190.4b |
|  | FE116 | 13.5 | 19.5 | 260.5a | 250.6a | 261.2a | 207.6ab | 191.9b | 192.4b |
|  | SD650 | 13.5 | 19.5 | 258.0a | 238.0a | 153.9b | 162.2b | 187.8b | 183.7b |
|  | MC670 | 13.5 | 19.8 | 237.7a | 222.8a | 192.7a | 198.6a | 192.3a | 210.0a |
|  | DH515 | 13.5 | 16.6 | 239.1a | 216.3a | 205.7ab | 207.6ab | 181.5ab | 147.3b |
| 2019 | DH551 | 13.5 | 20.1 | 230.1a | 214.0b | 215.0b | 212.0b | 209.6b | 188.5c |
|  | 909 | 13.5 | 20.0 | 278.6a | 192.1b | 180.9b | 180.7b | 150.0c | 143.1c |
|  | LP392 | 13.5 | 19.4 | 195.7a | 191.2ab | 183.3ab | 173.6bc | 172.0bc | 163.2c |
|  | FT5 | 13.5 | 17.9 | 207.3a | 167.4b | 166.1b | 139.7c | 178.8b | 121.9d |
|  | ND372 | 13.5 | 19.4 | 249.3a | 207.3b | 189.0b | 157.c | 149.4c | 136.6c |
|  | LP259 | 13.5 | 18.9 | 251.6a | 170.5b | 158.6b | 159.9b | 156.5b | 134.7c |
|  | SD660 | 13.5 | 19.4 | 185.4a | 178.9ab | 173.9ab | 168.9b | 164.0b | 145.2c |
|  | XY72 | 10.5 | 19.4 | 268.1a | 232.6b | 202.5c | 193.7c | 203.4c | 204.4c |
|  | DH111 | 10.5 | 20.3 | 269.0a | 238.2b | 196.2b | 190.8c | 201.9c | 199.7c |
|  | DH1769 | 13.5 | 20.2 | 183.6a | 183.9a | 174.0a | 181.0a | 178.9a | 147.0b |
|  | DD317 | 10.5 | 16.9 | 192.9a | 184.0ab | 179.2ab | 178.5ab | 172.3b | 173.8b |
|  | FD191 | 10.5 | 20.2 | 310.8a | 227.7b | 199.1c | 195.9c | 190.5c | 191.0c |
|  | XY99 | 13.5 | 19.5 | 300.4a | 171.5b | 166.1b | 175.3b | 169.1b | 157.0b |
|  | NH221 | 10.5 | 20.0 | 250.0a | 225.2b | 199.2c | 193.2c | 171.0d | 163.2d |
|  | XT1102 | 13.5 | 19.8 | 207.5a | 173.2ab | 175.5ab | 173.1ab | 176.5ab | 158.5b |
|  | LP312 | 13.5 | 18.6 | 250.2a | 181.9b | 165.8c | 164.9c | 154.8c | 154.9c |
|  | MC703 | 10.5 | 21.8 | 314.4a | 215.0c | 223.3bc | 198.9d | 232.4b | 213.3c |
|  | SD650 | 13.5 | 19.6 | 262.5a | 203.4b | 171.8c | 169.7c | 153.3c | 149.1c |
|  | MC670 | 13.5 | 20.0 | 253.5a | 206.4b | 200.5bc | 192.7bc | 178.7c | 146.1d |
|  | MC670 | 10.5 | 22.0 | 276.7a | 207.5b | 198.9b | 188.1b | 202.2b | 200.1b |
|  | XY77 | 10.5 | 20.1 | 210.7a | 194.3ab | 179.4b | 175.0b | 196.1ab | 173.9b |
|  | XY99 | 10.5 | 22.6 | 245.7a | 204.7bc | 217.0bc | 224.0ab | 197.0c | 167.2d |
|  | MC703 | 13.5 | 19.1 | 252.7a | 172.3b | 159.6b | 197.0b | 185.7b | 160.3b |
|  | DH1786 | 13.5 | 16.2 | 174.3a | 150.2b | 145.6b | 145.8b | 142.5b | 123.7c |
|  | LD575 | 10.5 | 19.7 | 210.9a | 210.5a | 208.4a | 207.3a | 206.6a | 185.3a |
|  | LD575 | 13.5 | 16.0 | 185.0a | 184.5a | 168.2ab | 157.3b | 154.2b | 126.8c |
|  | A316 | 13.5 | 14.7 | 242.7a | 171.9b | 159.6b | 159.7b | 165.4b | 116.3c |
|  | XY77 | 13.5 | 19.8 | 222.9a | 169.8b | 165.4b | 163.8b | 167.5b | 145.7c |
|  | DH1786 | 10.5 | 16.9 | 206.0a | 166.1b | 137.3c | 140.0c | 139.6c | 136.7c |
